# Spatial risk for a superspreading environment: Insights from six urban facilities in six global cities across four continents

**DOI:** 10.3389/fpubh.2023.1128889

**Published:** 2023-04-05

**Authors:** Becky P. Y. Loo, Ka Ho Tsoi, Kay W. Axhausen, Mengqiu Cao, Yongsung Lee, Keumseok Peter Koh

**Affiliations:** ^1^Department of Geography, The University of Hong Kong, Hong Kong, Hong Kong SAR, China; ^2^School of Geography and Environment, Jiangxi Normal University, Nanchang, China; ^3^Department of Civil, Environment and Geomatic Engineering, ETH Zürich, Zürich, Switzerland; ^4^School of Architecture and Cities, University of Westminster, London, United Kingdom

**Keywords:** pandemic, superspreading environment, public facilities, spatial risk, facility agglomeration, place-based strategy

## Abstract

**Introduction:**

This study sets out to provide scientific evidence on the spatial risk for the formation of a superspreading environment.

**Methods:**

Focusing on six common types of urban facilities (bars, cinemas, gyms and fitness centers, places of worship, public libraries and shopping malls), it first tests whether visitors' mobility characteristics differ systematically for different types of facility and at different locations. The study collects detailed human mobility and other locational data in Chicago, Hong Kong, London, São Paulo, Seoul and Zurich. Then, considering facility agglomeration, visitors' profile and the density of the population, facilities are classified into four potential spatial risk (PSR) classes. Finally, a kernel density function is employed to derive the risk surface in each city based on the spatial risk class and nature of activities.

**Results:**

Results of the human mobility analysis reflect the geographical and cultural context of various facilities, transport characteristics and people's lifestyle across cities. Consistent across the six global cities, geographical agglomeration is a risk factor for bars. For other urban facilities, the lack of agglomeration is a risk factor. Based on the spatial risk maps, some high-risk areas of superspreading are identified and discussed in each city.

**Discussion:**

Integrating activity-travel patterns in risk models can help identify areas that attract highly mobile visitors and are conducive to superspreading. Based on the findings, this study proposes a place-based strategy of non-pharmaceutical interventions that balance the control of the pandemic and the daily life of the urban population.

## 1. Introduction

Disease outbreaks can be triggered by superspreaders who infect a disproportionately larger number of people than would be suggested by the basic reproduction number (Rt) (1–4). From a public health perspective, identifying and isolating these patients in a timely manner can be critical in stopping the spread of an epidemic. This was one of the major lessons learned from the severe acute respiratory syndrome (SARS) outbreak in 2002–2004 (5). Beyond individuals, a superspreading environment can exist across space and time. While research on superspreaders focuses on people, a superspreading environment needs to consider the interactions of people, environment and the pathogen. Theoretically, an individual's reproductive number can be seen as a random variable representing the expected number of cases caused by a particular infected individual (6). The spatial risk factor is affected by the number and characteristics of visitors, and the functions and conditions of a place, among others. Identifying and closing down selected facilities can help stop the spread of an epidemic within a community by eliminating high-risk superspreading environments (7). However, focused research on spatial risk factors contributing to a superspreading environment has been limited. Moreover, the relationship is highly complex. In particular, a superspreading environment is not only caused by physical or environmental factors, such as ventilation and building design, but also a multitude of human factors, including the characteristics and activities of visitors. Medical factors, including and the transmission mechanisms and Rt, also play a crucial role.

With COVID-19, many governments have implemented non-pharmaceutical interventions to avoid overwhelming hospital and intensive care unit capacity during 2020–2022 (8, 9). While early interventions have proven to be effective in slowing down the disease, there are enormous socio-economic costs that hugely disrupt the economy and daily life of people (10, 11). So far, government interventions tend to follow a rather broad-brush approach based on complete lockdowns or the closure of public facilities by broad categories (12). Can a more differentiated approach be adopted? This study sets out to provide scientific evidence for more differentiated non-pharmaceutical interventions. Focusing on six common types of facilities (bars, cinemas, gym and fitness centers, places of worship, public libraries and shopping malls), it first tests whether visitors' mobility characteristics vary for different facility types and at different locations. Stemming from classical locational theories of Christaller and Losch, facility agglomeration is not only associated with external economies of scales but also different social mix of people (13). This pattern has been validated in a recent study about COVID-19 in Hong Kong (7). In order to establish evidence beyond one city, this study collects detailed human mobility data in six major global cities across four continents (Chicago, Hong Kong, London, São Paulo, Seoul and Zurich) to see whether the identified patterns are consistent. Again, as validated in the case of Hong Kong, although the historical mobility data do not cover the COVID-19 period, human mobility patterns demonstrate a high degree of temporal regularity which can provide very useful implications in epidemic prevention (14, 15). Methodologically, this study offers a novel approach that integrates human mobility, facility and activity data in identifying a superspreading environment. More broadly, the integration of mobility patterns in superspreading analysis is essential because a visitor who has a high travel intensity (a visitor making more trips and longer distances on the same day) is more likely to spread the disease. Just examining the density or volume of visitors does not take into account the disease spread risk based on the travel profile of visitors. The attempt of applying mobility data in understanding superspreading is, therefore, timely and needed.

## 2. Literature review

**Figure 1:**
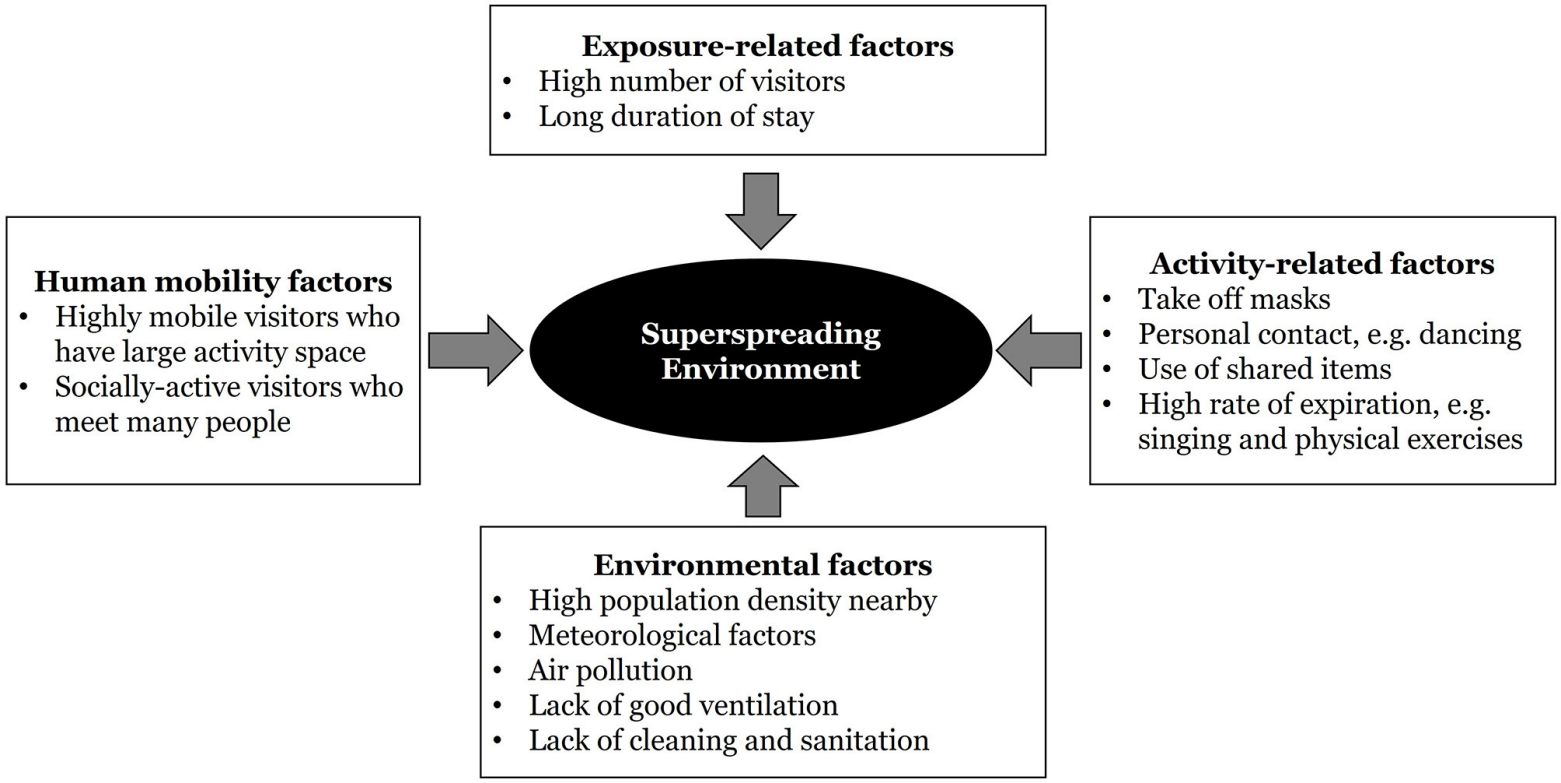
A schematic diagram of risk factors for the formation of a superspreading environment.

A superspreading environment exists when several conditions are met. Figure 1 shows these risk factors. The first group of factors relate closely to the absolute number of visitors at a location. As in safety analysis, the density and volume of vehicles and people directly increase the exposure of individuals to traffic crashes (16, 17). In the context of disease spread, the more visitors that a place has, the higher is the chance that one gets in touch with an infected individual, especially when asymptomatic individuals continue to carry out daily activities outside homes (18–20). In relation, many cities introduced non-pharmaceutical interventions, such as the banning of mass gatherings. In Hong Kong, a major indicator on the intensity of such interventions is the maximum group size allowed in restaurants; it ranged from 2 only during the tighter period to 30 during more relaxed time (21). These are the exposure-related factors in Figure 1.

The next group is human-mobility factors which capture the characteristics of people visiting a particular location (Figure 1). Some places attract visitors who are highly mobile and socially active. They carry out diverse activities by traveling and meeting a lot of people throughout the day. In contrast, some facilities may mainly be used by people who stay in their neighborhoods most of the time. A recent study uses a historical dataset on urban mobility to calculate the generalized activity space and the volume of space-time prisms of individuals in Hong Kong during a pre-pandemic period (7). It was found that the concentration of some facilities, such as bars, can greatly increase the chance of disease outbreaks as they tend to attract highly mobile and socially active patrons from far away. Yet, the lack of agglomeration can give rise to a highly mobile profile of visitors for other facilities. In other words, the nature and type of public facilities matter in explaining mobility characteristics of visitors and the formation of a superspreading environment (7, 22).

The third group is comprised of environmental factors (Figure 1). They include the physical settings of a place. Indoor places with poor airflow and ventilation are at risk (23). In particular, aerosol transmission is likely as respiratory droplets (diameter >5–10 μm) and droplet nuclei (diameter < 5 μm) tend to build up in closed spaces (24). Other meteorological conditions like temperature and humidity are relevant, with cold temperature combined with high humidity being more favorable to coronavirus survival and disease spread (25, 26). The chemical nature and concentration of particulate matters, as well as surface characteristics, also count (27). In an area where the population density is high and indoor environment with central air conditioning is common, the risk of disease spread also increases (28, 29).

The fourth group refers to activity-related factors (Figure 1). Places have different functions. Some activities that involve close physical contact (such as dancing) and taking off face masks (such as singing, eating, drinking and doing physical exercise) are high-risk (30, 31). The duration of engaging in these activities without face masks and without social distancing also matters (32, 33). Reports of irresponsible behavior of infected individuals, such as intentionally ignoring self-isolation, going to crowded places, as well as deliberately sneezing or coughing in public spaces and on common items, have raised concerns (1).

While these factors have been examined in isolation, it is only recently that a synthesis of the analysis in specific geographical settings and at a city scale has been conducted (7, 22, 34). This is the research gap that this study aims to fill. Can data on facility location, activities and human mobility be combined to identify a superspreading environment? In particular, is there any relationship between facility agglomeration and visitors' travel intensity? Would a concentration of facilities attract highly mobile visitors that further increases the chance of superspreading? By answering these questions, this research contributes to the literature of the spatial spread of diseases. Practically, identifying areas of higher superspreading risks can help governments to formulate place-based measures, such as closing selected public facilities at designated places rather than a uniform policy of complete lockdown or closing of all major public facilities in a non-discriminatory manner. With the COVID-19 pandemic, there are more focused studies on the value of non-pharmaceutical interventions in controlling disease spread and mitigating the impacts of the pandemic. One highly-relevant study evaluates the impacts of implementing confinement measures and mobility restrictions based on the calculation of the effective reproduction number (35). Other studies suggest that government interventions implemented in different periods of the pandemic, such as confinement, limiting geographical mobility and displacing high-risk passenger groups in public transport, were effective in controlling disease spread (36–38). Adopting more differentiated non-pharmaceutical interventions could help maintain a balance between public health risk and urban vibrancy, especially in global cities. It would also allow the urban population to maintain at least some social activities, such as eating out with friends, reading books in libraries, attending religious events, and doing physical exercise. Urban social life plays an important role in the psychological and physical wellbeing of the population.

## 3. Materials and methods

Following the daily routines in cities, we select six common types of urban facilities. Bars, shopping malls, cinemas, public libraries, and sports centers are also the points of interest (PoIs) examined in Loo et al. (7). However, as small shopping centers are difficult to define in a cross-country setting, only department stores and shopping malls are included in this international comparison. In addition, we also include places of religious worship due to the identification of disease clusters associated with religious places where the sharing of common items, such as bibles and food, is common (32). The detailed procedures are explained in Section 3.3.

### 3.1. Study areas

The six global cities selected in this study are Chicago in the United States, Hong Kong in China, São Paulo in Brazil, London in the UK, Seoul in South Korea and Zurich in Switzerland. The selection criteria are primarily based on geographical coverage (covering as many continents as possible), connectivity (Alpha cities in the Globalization and World Cities Research Network, GaWC, city classification), and the availability of disaggregate travel databases for detailed mobility analysis. These cities are in four different world regions, that is, Asia, Europe, North America and South America. The study area statistics, including the territorial size, population, GDP per capita, number of COVID cases, and number of COVID fatalities (up data available in May, 2022) are summarized in the Supplementary material.

### 3.2. Data

Three major types of data are used to conduct the analysis of superspreading environment. Table 1 summarizes the sources and information of the extracted datasets. Firstly, locational data were compiled. The data sources are the OpenStreet Map database and local authorities' geographical information system databases. We adopted the amenity classification from OpenStreet Map to extract the six types of PoIs in each city based on the amenity/shop/leisure tags. This is to maintain consistency across cities. To illustrate, we first used several keywords to identify the six types of PoIs, including “amenity = bar” for bars, “amenity = cinema” for cinemas, “leisure = fitness center” for gym and fitness centers, “amenity = place_of_worship” for places of worship, “shop = mall” for shopping malls, and “amenity = library” for public libraries. The extracted datasets were then further validated and checked manually. To illustrate, we have cross-validated the OpenStreet Map datasets for libraries against the official locations of public libraries from the respective government websites and spatial databases.

**Table 1:** Data sources.

**Cities**	**Human mobility datasets**	**Public facilities PoIs**	**Population data**
Chicago	*The 2018 My Daily Travel Survey* (HTS) Survey period: September 2018–December 2018 Spatial coverage: Northeastern Illinois Sample: 12,400 households; over 30,700 individuals.	Openstreet Map database; Chicago Data Portal	U.S. Census Bureau (39)
Hong Kong	*Travel Characteristics Survey 2011* (TCS2011) Spatial coverage: Hong Kong SAR Survey period: September 2011–January 2012 Sample: 32,000 households; over 58,000 individuals.	Openstreet Map database; GeoCommunity database	Population Census 2011 (40)
London	*London Travel Demand Survey* Survey period: 2015–2016 Sample: 8,000 households; over 18,000 individuals.	Openstreet Map database	2011 UK Censuses (41)
São Paulo	*The Origin and Destination Survey 2017* Spatial coverage: The Municipality of São Paulo Survey period: June 2017–October 2018 Sample: 32,000 households; over 86,000 individuals.	Openstreet Map database	The Origin and Destination Survey 2017 (42)
Seoul	*National Household Travel Survey of Korea 2016* Spatial coverage: South Korea Survey period: May, 2016 Sample: 219,686 households; 523,989 individuals. (for Seoul only 42,740 households; 103,032 individuals.)	Openstreet Map database (Seoul Open Data Plaza and other databases)	2016 Population and Housing Census (43)
Zurich	*MOBIS* (Mobility Behavior in Switzerland) Spatial coverage: Switzerland Survey period: September 2019–October 2020 Sample: 5,375 participants	Openstreet Map database; Open Data Zürich	Federal Statistical Office (44)

Secondly, human mobility data were extracted from travel surveys. These datasets generally record detailed travel and activity information (i.e., trip origins, trip destinations, trip purpose, travel time, and transport modes) covering at least one sample survey day. Travel data in Chicago, Hong Kong and São Paulo were extracted from the most updated large-scale travel diary surveys conducted by local authorities. The Zurich dataset was compiled from a research project by ETH Zurich that has applied the methods of both survey questionnaires and GPS tracking on mobile phones to record human travel behavior (45). Datasets of Chicago, São Paulo and Zurich used in this study were trimmed from a larger survey database. For instance, relevant travel records within the city of Chicago came from a bigger dataset that covers the region of North-eastern Illinois. Note that only data within the city areas were consolidated and analyzed. For most mobility datasets, data expansion factors are provided officially so we apply the data expansion factors before analysis. For those without expansion factors (i.e, Zurich and Seoul), we calculated the expansion factors based on the actual sociodemographic features (i.e., age groups and gender) and sociodemographic profiles of respondents in the survey. To conduct travel behavior analysis, network datasets were also extracted from respective government websites.

### 3.3. Methodology

**Figure 2:**
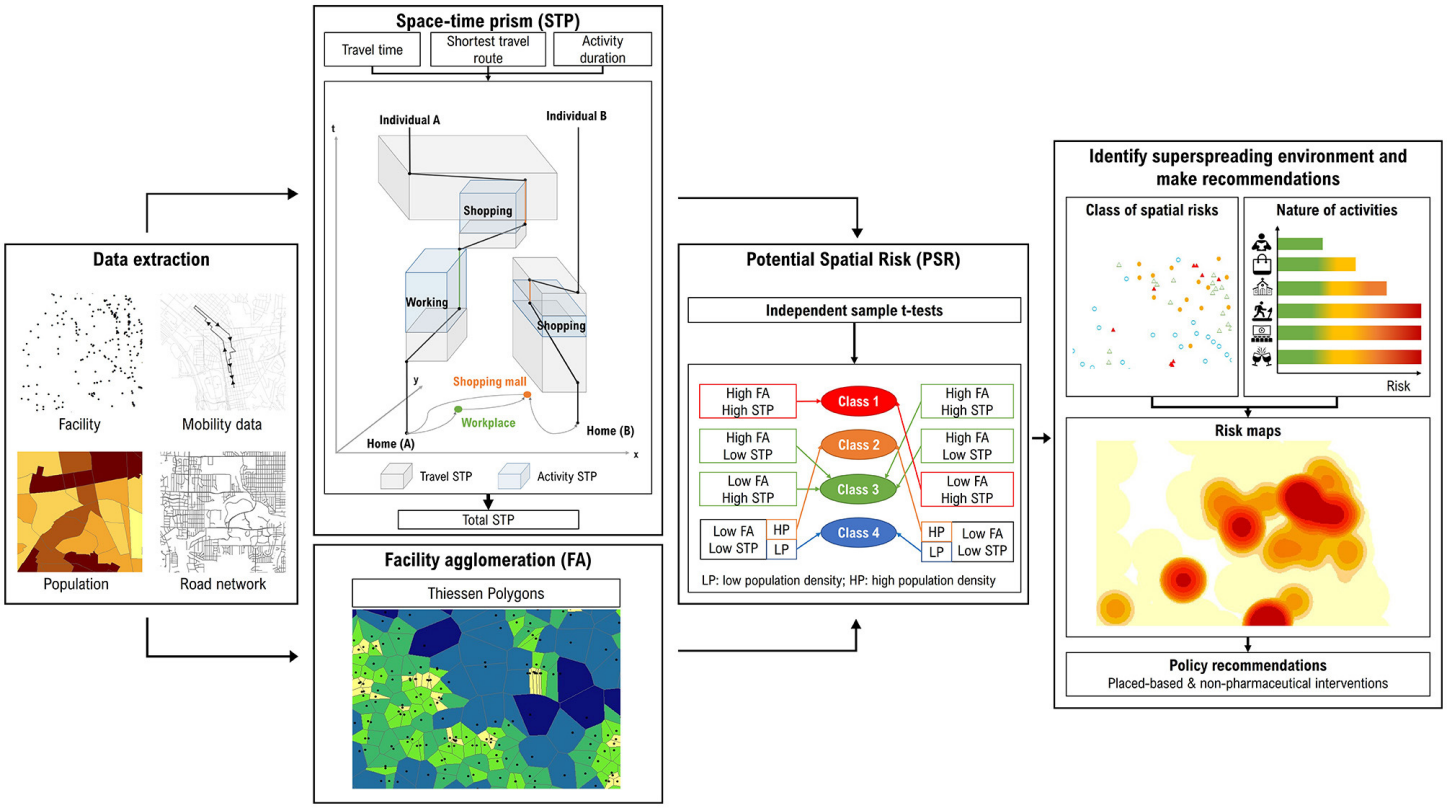
Methodological framework.

This paper makes use of the association between facility agglomeration and visitors' travel intensity to help determine the potential spatial risks for different PoIs; it then estimates the overall risk of superspreading in a city based on kernel density. Figure 2 illustrates the methodological framework. Firstly, we locate different types of urban facilities and capture individuals who have visited them from the six mobility datasets. Secondly, applying the individual travel data, we use the space-time prism (STP) methodology to measure visitors' travel intensity (section 3.3.1). In parallel, facility agglomeration is measured by a geometrical approach known as Thiessen polygons (section 3.3.2). Combining the two measures, we use the independent sample *t*-tests to test the association between the travel intensity of visitors and the level of facility agglomeration. Thirdly, a classification of spatial risk is developed based on the travel intensity, facility agglomeration, and population density nearby (section 3.3.3). Finally, integrating information about the spatial risk class and nature of activities at these facilities (30), we compile a risk map of superspreading environment (SE-risk map) for each city (section 3.3.4).

#### 3.3.1. Space-time constructs of samples visiting the PoIs

A first task is to examine the mobility or travel behavior of people visiting each facility. Individuals who have high travel intensity (notably those making more trips and longer distances) and are spending more time on out-of-home activities (whether mandatory, discretionary or subsistence) with a larger geographical coverage and over a longer time period are more likely to spread infectious diseases under the pandemic (7). To identify individuals visiting a targeted PoI, there are two steps. Based on the mobility datasets, we first find out individuals who visited the spatial unit where the targeted PoI is located. We then determine whether the trip purpose matched. For instance, a survey respondent who went to the spatial unit where a shopping mall is located and reported a trip purpose of “shopping” is considered to be visiting the shopping mall.

Next, we quantify the spatiotemporal extent of their daily activity-travel patterns. To do so, the extracted human mobility datasets are organized in a trip-based format, that is, each row is an origin-destination record (i.e., OD pairs) for an individual. For databases that were originally organized in a person-based format, they were first transformed by database restructuring. The travel intensity is measured by the STP methodology. Based on time geography, this methodology has been applied in previous studies to determine the level of travel and activities across space and time (7, 46). As shown in Figure 2, the volume of a travel STP is the product of a minimum bounding 2-dimensional activity space generated using the shortest network travel route of each trip and the associated travel time. An activity STP is a measure of the space-time volume of an individual's activity, such as work and shopping. Over a day, the volumes of travel and activity STPs can be summed up for each individual to calculate his/her total daily mobility (STP). A larger value of STP suggests that the individual takes part in out-of-home activities with a larger spatiotemporal extent. For further analysis, individuals with the top 25% of daily STP are considered as being highly mobile or having “large STP”.

#### 3.3.2. Analyzing the relationship between spatial agglomeration and potential spatial risk

Facility agglomeration is examined by the Thiessen Polygon method, which divides the area based on the distribution of point features (i.e., locations of PoIs). Each PoI is then featured with the size of divided polygons. More clustered PoIs (higher density) will have smaller polygons than those that are sparsely located (lower density). In this study, we follow the threshold of high agglomeration as in Loo et al. (7), such that for each type of PoI, the 25% with the smallest Thiessen polygons will be considered as “high agglomeration”, whereas others will be considered as “low agglomeration”.

As we ask whether there is an association between the degree of facility agglomeration and the travel intensity of people visiting them, we conduct an independent sample *t*-test to determine whether there is a statistically significant difference of the population means (i.e, STP) among two independent groups (i.e., low and high spatial agglomeration). The results of the compare-means test will lead to three possible theoretical outcomes: (i) the agglomeration (high density) of PoIs is associated with a larger STP (Group A), (ii) the lack of agglomeration (low density) of PoIs is associated with larger STP (Group B) and (iii) no statistical relationship between agglomeration and STP level (Group C). Results of this geographical analysis will inform our next step of assigning the spatial risk class based on the type of facility and the level of agglomeration.

#### 3.3.3. Defining PoIs based on the four potential spatial risk classes

Then, we define four potential spatial risk (PSR) classes based on facility agglomeration, travel intensity, and population density nearby. For Group A, since higher facility agglomeration is associated with a larger STP, highly-concentrated facilities are conducive to a superspreading environment (Class 1). On the contrary, for Group B, since the lack of agglomeration is associated with visitors with high mobility, low facility agglomeration is considered the most vulnerable (Class 1). As Class 1 is contributing much more to the risk of superspreading, we also test a more stringent threshold for facility agglomeration (12.5% or half of the 25% used throughout this study) in identifying Class 1 PoIs. For PoIs with low facility agglomeration and low visitor mobility, we further classify them into two sub-categories based on population density. If a PoI is located at a census unit of the top 25% of population density in the city, it is considered “high population density”. Facilities located within a densely populated area are vulnerable to localized outbreak (7). These areas are assigned Class 2. If population density, facility agglomeration and mobility of visitors are all low, the spatial risk is the lowest and these PoIs are classified as Class 4. Other combinations belong to Class 3.

#### 3.3.4. Generating the risk maps of superspreading environment

Finally, we generate the SE-risk map for each city. A kernel density function is employed to derive the risk surface for the six types of PoIs. Apart from the spatial distribution of facilities and population density, this study integrates two additional risk factors in estimating the superspreading risk—PSR class and nature of activities. The four PSR classes are weighted exponentially (i.e, 1, 10, 100 and 1,000) to illustrate the different risk levels. Different from Loo et al. (7), the nature of activities is also considered as a factor that affects the risk of disease spread. For instance, drinking at bars tends to be more prone to disease spread than reading books at libraries, as the former usually involves people taking off masks and having louder conversations. Hence, the six types of facilities are weighted with a scale of 1–5 by referring to the Risk Assessment Chart (30) that ranks different activities on various risk levels. Accordingly, we assign a weight of 5 to bars, cinemas, and gym & fitness centers; 4 to places of worship; 3 to shopping malls; and 2 to public libraries. Integrating the above factors and using the kernel density function, a risk map is generated to illustrate the potential risk surface for each city. In general, the higher the kernel density values, the higher the superspreading risk. The kernel density values are displayed in eight equal quantiles for better visualization. Areas with the top 25% values in the city (i.e., the 75th percentile) are defined as “high-risk”.

## 4. Results

### 4.1. Descriptive statistics

Table 2 depicts the descriptive statistics for the number of facilities, mobility characteristics of visitors, number of samples, and population after data expansion in the six global cities. The total number of PoIs (visited by respondents in the travel surveys) across cities ranges from 235 in Hong Kong to 978 in Seoul. In general, there is a comparable number of PoIs among several common types of facilities including cinemas, public libraries, and shopping malls (typically with each accounting for < 10% of total PoIs in a city). Although the number of PoIs visited varies across different cities, the highest proportion of facilities visited is bars. The share varies from 29.42% in London to 63.04% in Chicago. Gym rooms in Hong Kong and Seoul got fewer reported visits (5.96 and 4.81%, respectively) possibly due to cultural differences that Asians tend to go to gyms and fitness centers less often. They were more popular in London (22.76%) and São Paulo (16.65%). Places of worship were well frequented in Seoul (46.32%), London (29.85%), São Paulo (28.65%) and Hong Kong (28.09%).

**Table 2:** Descriptive statistics.

**PoIs**	**Chicago**	**Hong Kong**	**London**	**São Paulo**	**Seoul**	**Zurich**
**Bars**						
No. of PoIs	232	96	411	143	306	308
Mean STP	350.80	197.28	74.42	98.04	45.61	92.69
Std. of STP	740.10	419.44	118.33	202.12	81.25	275.37
Samples visiting the PoIs	2,136	195	914	373	2,834	2,518
Population after data expansion	417,178	12,708	429,090	40,566	70,751	411,521
**Cinemas**						
No of PoIs	11	28	66	19	49	20
Mean STP	376.10	204.19	127.53	49.25	29.88	121.15
Std. of STP	521.02	322.51	436.52	74.96	69.69	529.03
Samples visiting the PoIs	83	69	433	120	2,225	462
Population after data expansion	19,681	42,56	187,849	13,721	61,526	136,417
**Gyms and fitness centers**						
No of POIS	33	14	318	59	47	52
Mean STP	465.51	67.79	120.78	63.45	17.61	160.79
Std. of STP	794.42	91.66	478.59	89.54	48.86	528.22
Samples visiting the PoIs	230	30	733	287	8,215	672
Population after data expansion	39,727	5,876	328,431	34,510	236,785	135,890
**Places of worship**						
No of PoIs	34	66	417	108	453	106
Mean STP	776.75	210.77	16.88	52.27	39.55	62.85
Std. of STP	1,127.48	385.30	56.24	90.27	85.38	184.36
Samples visiting the PoIs	46	93	496	301	1,791	635
Population after data expansion	16,286	13,468	210,576	46,565	50,284	148,306
**Public libraries**						
No of PoIs	31	20	112	25	100	19
Mean STP	465.22	80.25	31.76	85.92	22.52	50.83
Std. STP	1,034.10	137.56	79.24	149.18	63.73	170.19
Samples visiting the PoIs	85	68	198	157	4,649	270
Population after data expansion	23,353	4,292	95,898	22,919	13,3873	68,630
**Shopping malls**						
No of PoIs	27	11	73	23	23	15
Mean STP	287.80	83.07	14.34	121.15	50.08	72.63
Std. of STP	554.09	152.36	140.32	112.81	132.08	297.10
Samples visiting the PoIs	405	895	2,928	54	1,070	1,200
Population after data expansion	94,776	57,450	1,336,250	8,927	29,285	223,033
**Total**						
No of PoIs	368	235	1,397	377	978	520
Mean STP	365.03	119.63	46.88	73.72	27.07	94.18
Std. of STP	743.48	256.68	240.40	136.05	69.69	347.56
Samples visiting the PoIs	2,985	1,350	5,702	1,292	20,784	5,757
Population after data expansion	611,002	98,049	2,588,094	1,67,208	583,045	1,123,798

Regarding the size of daily STP, the highest average was found in Chicago (>360 km^2^h), which may be attributed to the higher mobility and car-oriented culture in the United States. To recall, the STP is calculated based on the spatial extent of an individual's activity and travel space and the duration spent at a location. The other five cities were having a lower level of daily mobility (STP < 120 km^2^h). While analyzing the factors of affecting STP is not the major focus of this paper, some research has examined the impacts of built environment (e.g., facility density and accessibility to opportunities), trip characteristics (e.g., mode choices), and activity patterns on STP (47, 48).

Across different facility types, people traveling to places of worship and bars typically had larger STP in Chicago (776.75 and 350.8 km^2^h, respectively) and Hong Kong (210.77 and 197.28 km^2^h, respectively). The STP was substantially lower in London (16.88 and 74.42 km^2^h, respectively), São Paulo (52.27 and 98.04 km^2^h, respectively), Seoul (39.55 and 45.61 km^2^h, respectively) and Zurich (62.85 and 92.69 km^2^h, respectively). On the contrary, people visiting shopping malls had higher daily STP in Chicago (287.8 km^2^h) and São Paulo (121.15 km^2^h) but not in London (14.34 km^2^h) and Seoul (50.08 km^2^h). All these observations reflect the geographical and cultural context of various facilities, transport characteristics and people's lifestyle observed across cities.

### 4.2. Spatial agglomeration and individual daily STP

**Figure 3:**
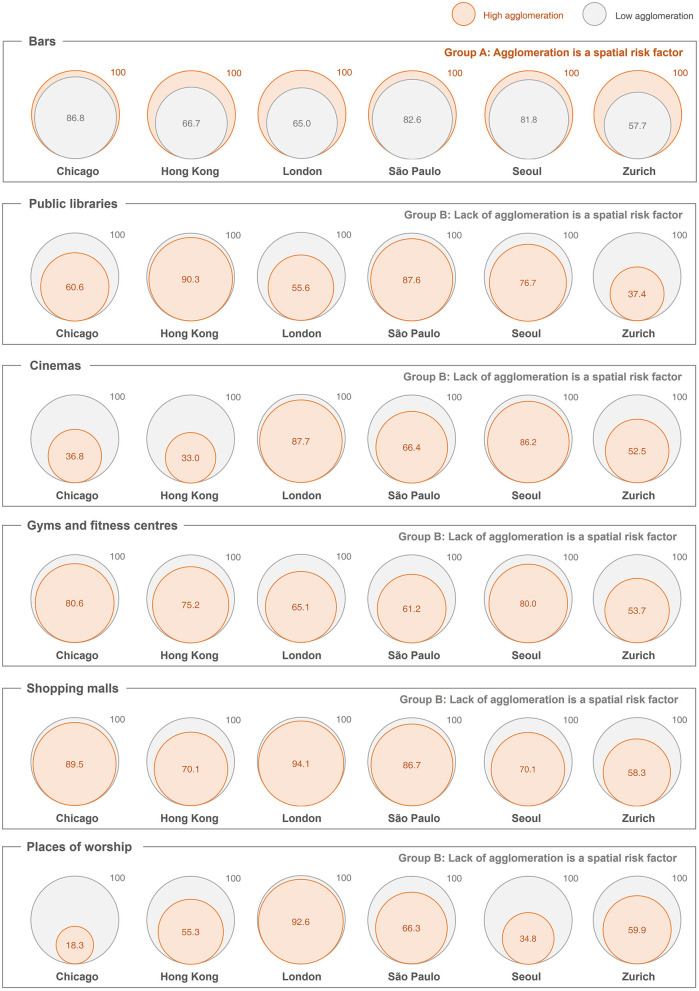
Space-time volume of public facilities with different levels of spatial agglomeration. Note: The prisms indicate the average volume of daily STP (km^2^h). Difference-of-means tests are conducted for the low-density and high-density PoIs in the six selected cities. All are statistically significant difference at p<0.05 level. For better illustration and comparison within a city, the size of STP is normalized that the higher STP (for low-density or high-density) in a city for a facility is set to an index of 100.

Based on the two groups of high and low facility agglomeration, Figure 3 summarizes the different mobility characteristics for visitors and results of the difference-of-means tests. To illustrate the magnitude of difference between the two groups, the values are normalized to 100 based on the STP of the group with a bigger value. Consistent with the findings in Hong Kong, geographical agglomeration is a risk factor for bars (Group A). In other words, the average STP of visitors in bars of high agglomeration (i.e., orange circles) is statistically higher than those of low agglomeration (i.e., gray circles) in all six cities. This indicates that an agglomeration of bars tends to attract patrons of higher travel intensity, which in turn enhances the spatial risk of superspreading. For other PoIs, the lack of agglomeration is a risk factor (Group B). Take gyms as an example, the average STP of visitors in less agglomerated gyms (i.e., gray circles) is statistically higher than those of highly agglomerated (i.e., orange circles). This suggests that “standalone” gyms tend to attract visitors of higher travel intensity and may potentially lead to higher superspreading risk. The results suggest that there is a clear association between facility agglomeration and travel intensity, but the relationship depends on the type of facilities.

### 4.3. Potential spatial risk of six types of PoIs in six global cities

**Figure 4:**
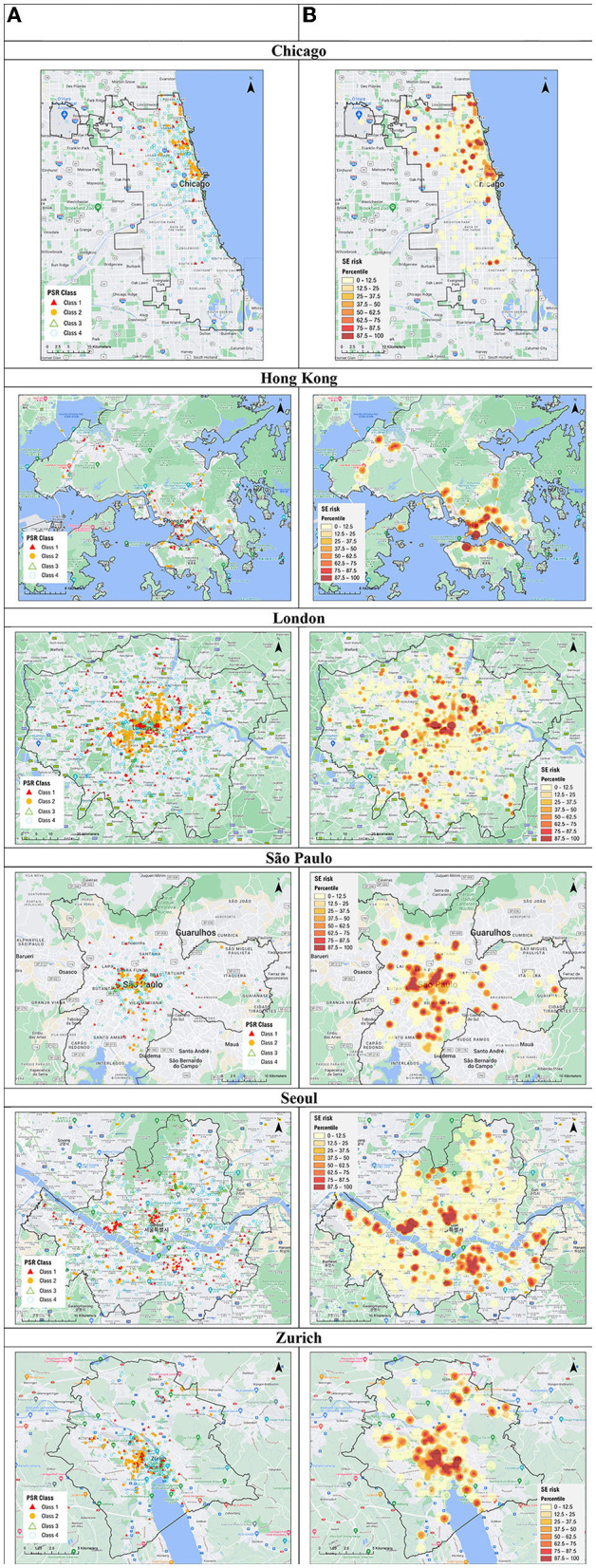
Maps of superspreading environment in six global cities. **(A)** Public facilities by potential spatial risk (PSR). **(B)** Risk map of superspreading environment (SE-risk map).

Following our analysis, Figure 4 shows the pattern of spatial risk at the city level. The maps on the left illustrate the distribution of public facilities by PSR classes. Among the Class 1 facilities, the shares of bars, public libraries, cinemas, shopping malls, gym and fitness centers, and places of worship are 30.1, 9.0, 5.3, 5.1, 18.9, and 31.5%, respectively. Before delving into the city-level analysis, we further compare our findings with a simpler approach of only considering PoI density. The PoI density maps are provided in the Supplementary Figure 1. When comparing both maps in each city, it is clear that the spatial patterns are not the same. Notably, many areas of low PoI density are actually having high superspreading risk when human mobility data are considered. Methodologically, integrating activity-travel patterns in risk models can enrich and supplement traditional methods (e.g., based on PoI density only). To test the robustness of our modeled results, we also tested another threshold of the top 12.5% of facility agglomeration as “high agglomeration”. The corresponding SE-risk maps are also provided in the Supplementary Figure 2. When the SE risk maps using the two thresholds are compared, the change does not affect the overall spatial patterns of SE risks. In most cases, the high-risk areas identified in the maps using the 25% threshold are still spotted in the ones using the 12.5% threshold. In the following paragraphs, we move on to the city-level discussion.

In Chicago, the highest superspreading risk can be found in Downtown Chicago, which is composed of the community areas of Chicago Loop (downtown area) and Near North Side. Both areas, in particular River North, are found with abundant recreational activities including bar clusters and some cinemas. Another high-risk cluster is the north of the downtown, which is near Lincoln Park and Lakeview community areas which are featured by night life and bars. The northeastern part of the city (i.e., Rogers Park) is a community with a university nearby and several beaches along the coast, where bars and cinemas are found. For other hotspots of superspreading risk, they are mainly associated with scattered places of worship, public libraries, shopping malls that attract highly-mobile visitors.

In Hong Kong, areas of the highest superspreading risk are mainly identified in the central business district (CBD) and urban cores. CBD is a term used in urban planning and management to describe the “core” of a city with an agglomeration of business establishments and services (49, 50). Central district is located in the CBD with office buildings; and there is also a famous bar cluster (i.e., Lan Kwai Fong). The urban core in Hong Kong (i.e., Tsim Sha Tsui) is also a major hotspot where clusters of bars and shopping malls are found. Several new towns in the northwest and northern part of Hong Kong (i.e., Tuen Mun, Yuen Long, Tseun Wan and Sha Tin) are also observed with high superspreading risk. These areas are mainly for residential use, where limited PoIs in the neighborhood (e.g., shopping malls or gyms and fitness centers) can attract visitors from far away. Also, some places with high population density (like Kwai Fong) have high potential risk of localized outbreak. It is observed that the bar clusters in Lan Kwai Fong and several types of PoIs with a lack of agglomeration located near new towns were associated with superspreading events (7, 51).

In London, areas with high PSR are concentrated in central and Inner London, particularly within zone 1, such as in the City of London, and the Boroughs of Westminster, Camden and Kensington and Chelsea. Many places of interest (e.g., Big Ben, Sherlock Holmes Museum), Soho (bars, cinemas), national library (the British Library), department stores (Harrods, Selfridges), and place of worship (Westminster Abbey, St. Paul's Cathedral) are all located in central London, where people tend to gather. Furthermore, there were a few superspreading points unevenly distributed in Outer London across different London Boroughs. Further research could also investigate, for instance, specific socially deprived and/or displacement areas in Greater London (52–55), which may exacerbate the possibility of a higher rate infection transmission (56).

In São Paulo, higher superspreading risk is clearly observed in the downtown area (i.e. Zone Central). Some sub-districts of Bela Vista and República are also characterized by a wide range of bars and clubs that may contribute to a superspreading environment. Moreover, the area is observed with other recreational PoIs such as cinemas and shopping malls near the metro station of República. Two other districts (i.e., Perdizes and Sumaré) are mainly residential areas where a wide variety of PoIs, including a larger number of places of worships, a library, some gym centers and a cinema, can be found. Finally, the scattered hotspots of superspreading are typically places of worship and gym centers located across the outskirt of the city.

In Seoul, superspreading environment is found around major transport hubs in old and new CBDs, Jongro in the middle and Gangnam in the southeast, and a few other secondary employment centers spread across space. In addition, many subway stations along the subway lines connecting these downtowns and employment centers present high potential infection risk. Like many other cities around the world, bars, movie theaters, and shopping malls are in the CBDs and employment centers to better serve customers who look for places for social and recreational activities after work. Not surprisingly, over time these PoIs also serve visitors from outside, who do not necessarily work nearby but find these PoIs more appealing than others. Note also that communities along those subway stations provide a great number of high-rise condominiums (i.e., “apartments”), which have been built rapidly since the early 1970s in response to rapid population growth, housing shortage, a lack of modern infrastructure, and slums. These hyper-dense residential areas have been created first in Gangnam (i.e., the southern half of Seoul) and later spread to Gangbuk (i.e., the northern half of Seoul). Again, localized outbreaks at these localities are likely. Our results also align with the empirical superspreading clusters in the Guro-gu district and the nightclubs clusters in Itaewon (57).

In Zurich, the major superspreading environment is District 4 (Aussersihl), which is a focal point for relaxation and night life in the entire city. It is a touristy area that features a cluster of bars, clubs, restaurants, as well as hotels. In addition, the traditional old town of the city (Altstadt) is a hotspot for disease spread, where some bar clusters and places of worships are located. Moreover, District 5 (Industriequartier), a neighborhood under gentrification, is a high-risk area where several bars and fitness centers are found. Finally, a local hotspot is found in District 11 (north of the city), which is a mixed residential and commercial area with some shopping malls, libraries, cinemas and fitness centers located. Empirically, one of the earlier superspreading events happened at a night club in District 5 (58).

## 5. Policy implications and conclusions

Based on the spatial risk maps, some high-risk areas of superspreading are identified across six global cities in four continents. As there is increasing evidence to show that COVID-19 is characterized by overdispersion in transmissibility that propels superspreading (3), our findings can be useful for city governments to formulate a more elaborate action plan during COVID-19, especially when a consistent or sudden increase in the number of cases is detected. Methodologically, our approach that integrates mobility data can identify areas that attract highly mobile visitors and are conducive to superspreading. As the pathogen is carried by human beings, the incorporation of human mobility data in risk mapping enriches and supplements more traditional approaches (e.g., PoI density) of disease risk mapping. Apart from facility agglomeration and travel intensity, other factors of superspreading environment, such as sociodemographic features (i.e., different age groups), social interactions, centrality of PoIs, and micro-environment of the PoIs would also need to be considered as further methodological refinements.

As the number of COVID-19 cases increases, it is recommended that the government can take rapid actions to adopt a place-based strategy of non-pharmaceutical interventions by locating areas with high risk of a superspreading environment in city and implement possible interventions to those PoIs with PSR Class 1 and Class 2. This can be done relatively quickly within a week that coincides with the onset of a new wave of the pandemic. This avoids the more drastic non-pharmaceutical interventions of a complete city lockdown or closing all facilities in a non-discriminatory manner throughout the city, as commonly practiced during the early pandemic (8, 9, 12, 35–38). Policy-makers, being better informed by the superspreading risk, can consider more targeted and place-based measures to suppress the formation of a superspreading environment. However, as for all non-pharmaceutical interventions, the implications, especially those on the vulnerable and disadvantaged groups of society, should be carefully considered (10, 11). As demonstrated in this study, though some common trends were identified, both the travel intensity (as measured by STP) and the level of facility agglomeration varied a lot among the six global cities. In addition, people's responses to government measures may also vary significantly (59–61). For instance, one recent study analyses activity patterns upon different COVID-19 measures (e.g., facility closures) among 149 countries (60). Though the measures introduced were targeted at specific facilities (rather than area-based, as suggested in this paper), the study found strong variations in the responses among grocery, park and work visits across different countries. Hence, the potential consequences of any non-pharmaceutical intervention, and how people respond to those measures are context-specific. Further in-depth research is warranted.

In the medium term, the physical settings of PoIs with PSR Class 1 should be improved with higher requirements of air ventilation and hygiene measures, as well as other tracing mechanisms, to lower the risk of superspreading. In the longer term, there are also planning implications for cities. Given that a lack of spatial agglomeration can give rise to a highly mobile profile of visitors to public facilities such as shopping facilities, karaoke/cinemas, public libraries, and sports centers. City administrators and planners should aim to further decentralize such facilities and encourage the creation of smaller, neighborhood sports centers and libraries to avoid residents having to travel far to use these basic facilities.

As the virus mutates, it can become more infectious. Recent evidence further suggests that the emergence of SARS-CoV-2 variants is facilitated by superspreading events (62). This has happened with the OMICRON variants that are much more contagious than those with which the pandemic began in 2020 (63). Under these circumstances, the virus is much more effective at creating a superspreading environment. The density of the population, activity type, facility agglomeration and visitors' profile are all significant factors in the spread of the disease. The failure to identify and close down a superspreading environment, for instance, within a restaurant or a housing estate, in a rapid manner can lead to large-scale local outbreaks. The failure of the Hong Kong SAR Government to isolate and enforce confinement at the Kwai Tsing Estate, where many confirmed cases were concentrated and first identified before the Chinese New Year 2022, was likely to have been a major reason for the eventual large-scale outbreak during the Fifth Wave of the pandemic in Hong Kong. This study underscores the importance of monitoring and controlling superspreading environment in relation to disease outbreak management.

As pointed out by Lloyd-Smith et al. (6), individual-specific control measures rather than population-wide measures can be much more effective in controlling disease outbreak. Similarly, the implementation of lockdown measures or closure of all public facilities of the same type is a type of population-wide measures that can be improved by informed superspreading environment analysis and location-specific control measures. Our analysis widens the investigation from a pure medical science focus to the environment, encompassing functions of places, location agglomeration, population density and human mobility patterns. Moreover, the empirical evidence generated can facilitate a better calibration and further refinements of theoretical models, such as the susceptible-infected-recovered and agent-based approaches [e.g., (4, 63, 64)]. It is recognized that the travel behavior and spatial agglomeration of PoIs might have changed before, during, and after the pandemic periods. Hence, future research can examine the dynamic relationship among travel characteristics, spatial agglomeration and the formation of superspreading environment based on real-time travel data (e.g., GPS trajectories of visitors). Future research can delve into the relationship between STP and other environmental variables. If mobility data of individuals infected by COVID-19 (e.g., travel diaries and GPS trajectories) are available, it will be feasible to conduct a more rigorous validation of the SE risk maps. Finally, further research directions include adding spatial heterogeneity to epidemic models that consider the people-environment interaction in a more holistic manner.

## Data availability statement

The data analyzed in this study is subject to the following licenses/restrictions: Some mobility data used to support the findings in this study are not publicly available because the datasets are under a license from the data provider. Requests to access these datasets should be directed to KT, kahotsoi@hku.hk.

## Ethics statement

Ethical review and approval was not required for the study on human participants in accordance with the local legislation and institutional requirements. Written informed consent for participation was not required for this study in accordance with the national legislation and the institutional requirements.

## Author contributions

BL: conceptualization, investigation, and supervision. KT: data curation, formal analysis, validation, and visualization. BL and KT: methodology and writing the original draft. BL and KA: project administration. MC, KA, YL, and KK: resources. All authors contributed to review and edit the manuscript, as well as to approve the submitted manuscript.
