# Supplementary material for: Spatial risk for a superspreading environment: Insights from six urban facilities in six global cities across four continents
Source: Front Public Health. 2023 Apr 5;11:1128889. doi: 10.3389/fpubh.2023.1128889 (PMC10113652; doi:10.3389/fpubh.2023.1128889)
Supplement: Supplementary file 1 [file Data_Sheet_1.docx]

Supplementary Material

Spatial risk for a superspreading environment: Insights from six urban facilities in six global cities across four continents

Becky P. Y. Loo^1,2^, Ka Ho Tsoi^1*^, Kay W. Axhausen^3^, Mengqiu Cao^4^, Yongsung Lee^1^, Keumseok Peter Koh^1^

^1^ Department of Geography, The University of Hong Kong, HKSAR, China

^2^ School of Geography and Environment, Jiangxi Normal University, China

^3^ Department of Civil, Environment and Geomatic Engineering, ETH Zürich, Switzerland

^4^ School of Architecture and Cities, University of Westminster, The United Kingdom

*** Correspondence:**Ka Ho Tsoi

kahotsoi@hku.hk

# Supplementary Tables

**Table S1. Study area statistics.**

| **Cities** | **Total area (km²)** | **Population** | **GDP per capita (US$)** | **COVID-19 cases** | **COVID-19 deaths** |
| --- | --- | --- | --- | --- | --- |
| Chicago, USA | 606.1 | 2,716,000 | 61,170^#^ | 600,474 | 7,689 |
| Hong Kong, China | 1,113.8 | 7,510,000 | 48,639 | 1,209,397 | 9,365 |
| London, UK | 1,572 | 9,426,000 | 67,741 | 2,890,115 | 23,259 |
| São Paulo, Brazil | 1,521.1 | 12,396,372 | 12,400 | 1,058,895 | 42,299 |
| Seoul, Korea | 605.2 | 9,736,027 | 38,489 | 3,577,930 | 4,770 |
| Zurich, Switzerland | 87.9 | 428,700 | 81,023* | 635,129* | 1,799* |

Notes: *Canton-wide data.

Sources: (1-17)

# Supplementary Figures

| **Chicago** | **Hong Kong** |
| --- | --- |
| 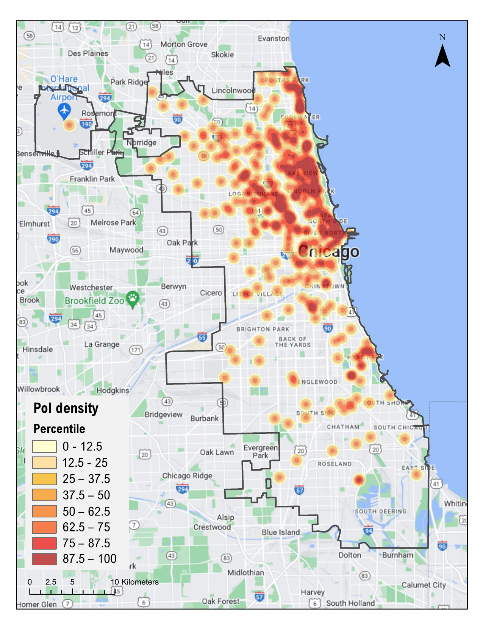 | 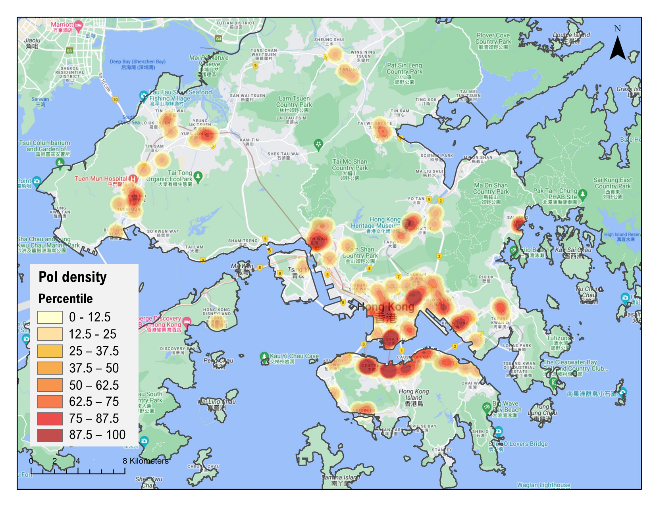 |
| **London** | **São Paulo** |
| 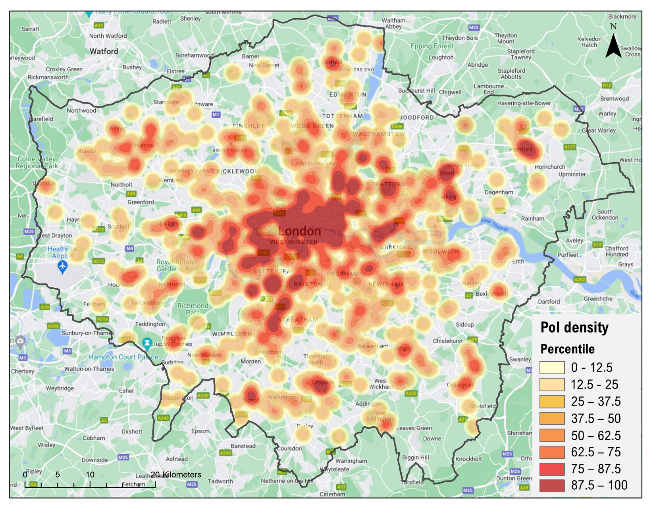 | 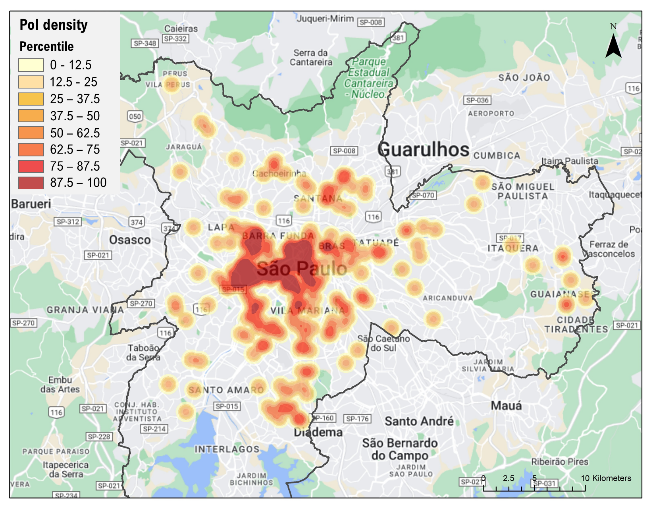 |
| **Seoul** | **Zurich** |
| 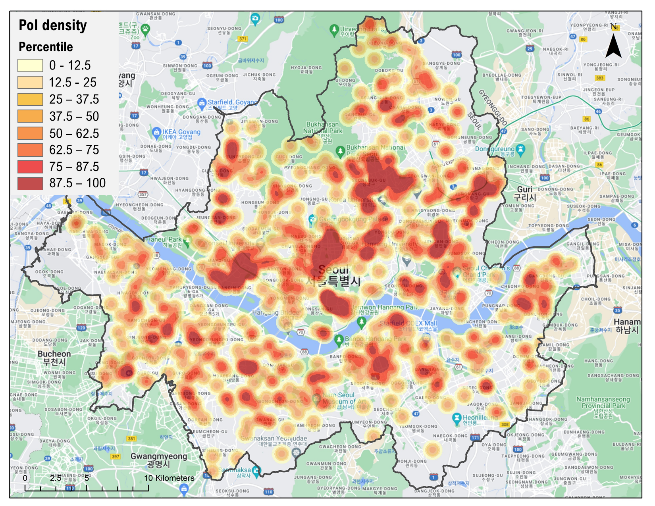 | 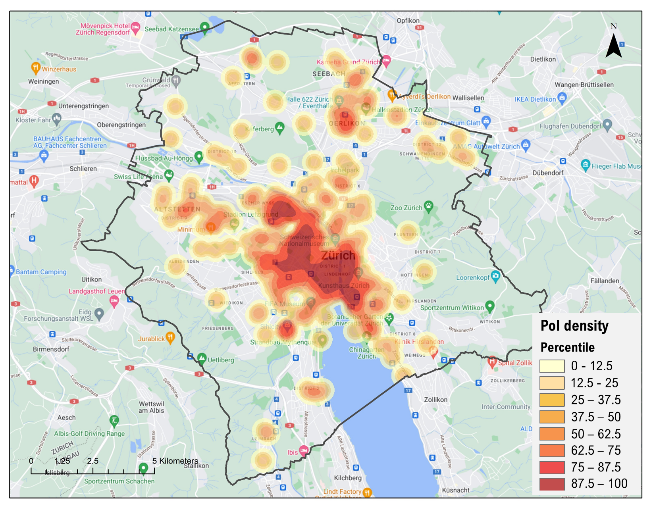 |

**Figure S1. PoI density maps in the six cities**

| **Chicago** | **Hong Kong** |
| --- | --- |
| 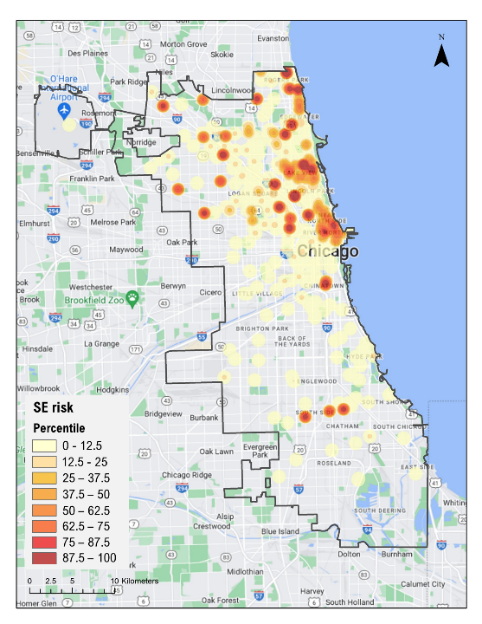 | 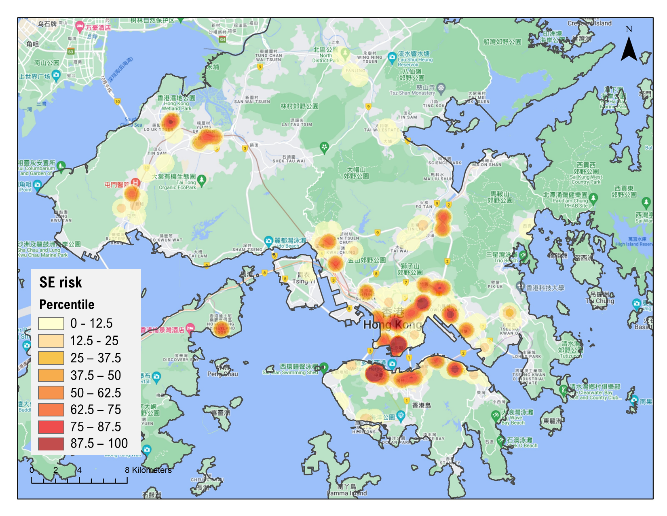 |
| **London** | **São Paulo** |
| 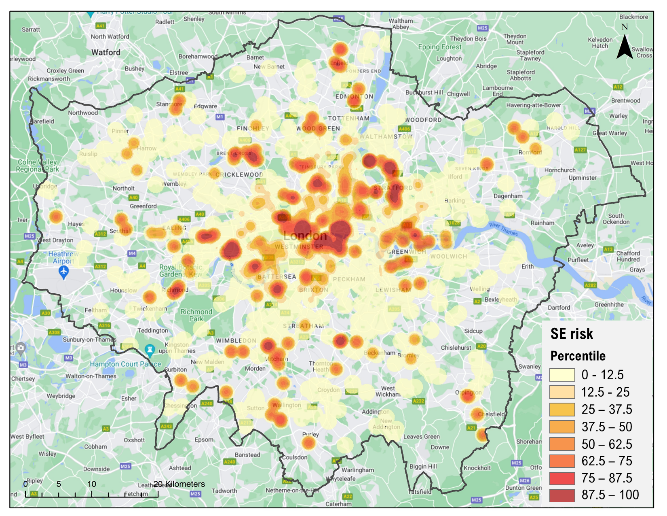 | 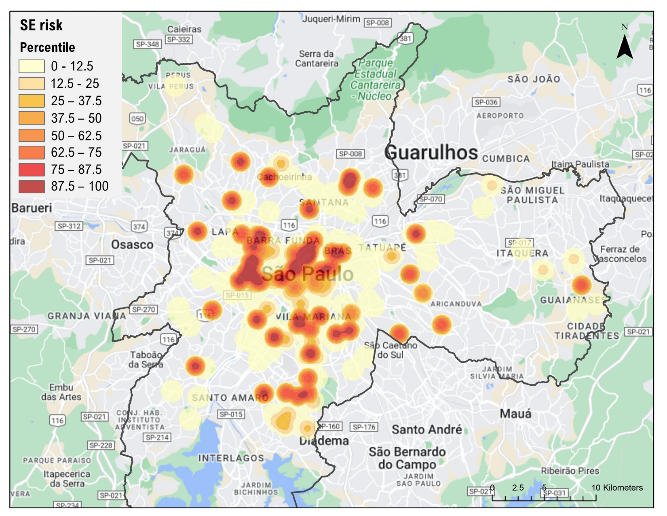 |
| **Seoul** | **Zurich** |
| 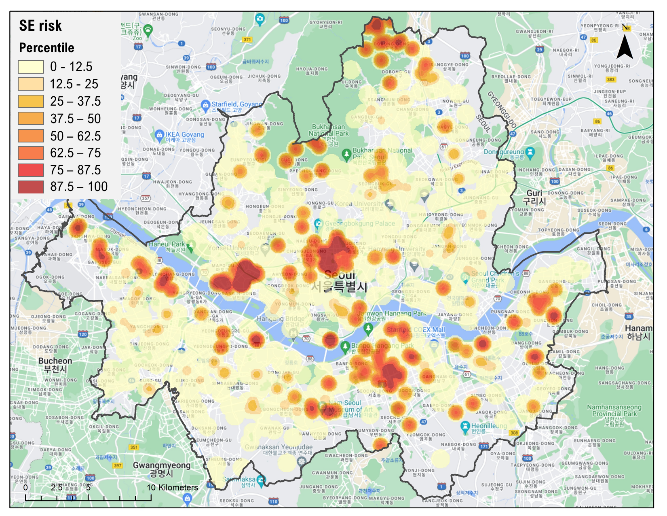 | 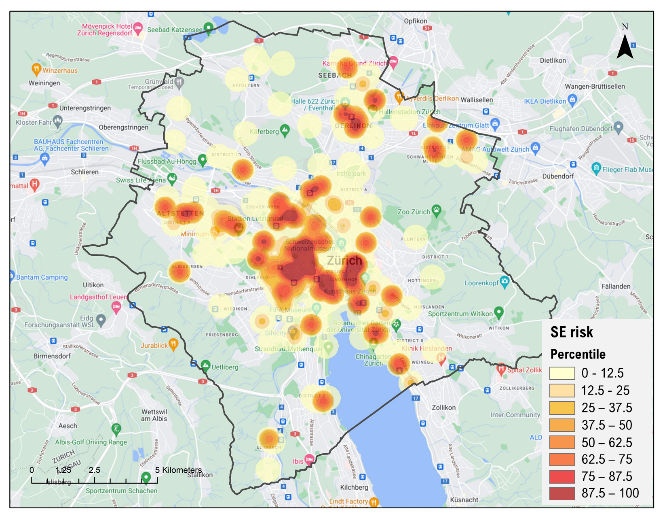 |

**Figure S2. Risk maps of superspreading environment (SE-risk map) using the threshold of the top 12.5% of facility agglomeration as “high agglomeration”.**

**References**

1. City of Chicago. *Facts & Statistics*. (2022a). Available online at: https://www.chicago.gov/city/en/about/facts.html (accessed 19 May 2022).
2. U.S. Bureau of Economic Analysis. *GDP & Personal Income*. (2022). Available online at: https://apps.bea.gov/iTable/index_regional.cfm (accessed 19 May 2022).
3. City of Chicago. *Chicago COVID-19 Summary*. (2022b) Available online at: https://app.powerbigov.us/view?r=eyJrIjoiYjUwNjEwN2QtYmJkYS00MTZmLTg4YjMtZGRkMzEzMmFmYjg4IiwidCI6IjcwMzZjZGE5LTA2MmQtNDE1MS04MTQ0LTk3ZGRjNTZlNzAyNyJ9 (accessed 19 May 2022).
4. GovHK. *Hong Kong – the Facts*. (2022) Available online at: https://www.gov.hk/en/about/abouthk/facts.htm (accessed 19 May 2022).
5. HKSAR Government. *Coronavirus Disease (COVID-19) in HK*. (2022) Available online at: https://chp-dashboard.geodata.gov.hk/covid-19/en.html (accessed 19 May 2022).
6. Macrotrends. *London, UK Metro Area Population 1950-2022*. (2022). Available online at: https://www.macrotrends.net/cities/22860/london/population (accessed 19 May 2022).
7. UK Office for National Statistics. *Regional economic activity by gross domestic product, UK: 1998 to 2018*. London: UK Office for National Statistics (2022).
8. GOV.UK. *Coronavirus (COVID-19) in UK*. (2022) Available online at: https://coronavirus.data.gov.uk/details/deaths?areaType=region&areaName=London (accessed 19 May 2022).
9. Portal do Governo Brasileiro IBGE. *São Paulo*. (2022). Available online at: https://cidades.ibge.gov.br/brasil/sp/sao-paulo/panorama (accessed 19 May 2022).
10. Portal do Governo Brasileiro Censo Agro 2017. *Editoria: Produto*. (2022) Available online at: https://censoagro2017.ibge.gov.br/en/2185-news-agency/releases-en/32601-city-of-sao-paulo-concentrates-10-3-of-brazilian-gdp-in-2019.html (accessed 19 May 2022).
11. International Association of Providers of AIDS Care. *São Paulo*. (2022) Available online at: https://www.fast-trackcities.org/data-visualization/s%C3%A3o-paulo-covid (accessed 19 May 2022).
12. Seoul Solution. *The Statistic of Seoul*. (2022). Available online at: https://www.seoulsolution.kr/en/content/statistic-seoul (accessed 19 May 2022).
13. Seoul Metropolitan Government. *COVID-19*. (2022) Available online at: https://www.seoul.go.kr/coronaV/coronaStatus.do (accessed 19 May 2022).
14. Statistics Korea. *Regional Income*. (2022) Available online at: https://kostat.go.kr/portal/korea/kor_nw/1/13/2/index.board?bmode=read&aSeq=387088 (accessed 19 May 2022).
15. StadtZürich. *Facts & Figures*. (2022). Available online at: https://www.stadt-zuerich.ch/portal/en/index/portraet_der_stadt_zuerich/zahlen_u_fakten.html (accessed 19 May 2022).
16. Bundesamt für Statistik. *Gross domestic product (GDP) per region and canton*. (2022). Available online at: https://www.bfs.admin.ch/bfs/de/home/statistiken/volkswirtschaft

/volkswirtschaftliche-gesamtrechnung/bruttoinlandproduktkanton.assetdetail.10647599.html (accessed 19 May 2022).

1. Opendatasoft. *COVID-19 Pandemic - CH/Switzerland*. (2022). Available online at: https://public.opendatasoft.com/explore/dataset/covid-19-pandemic-chswitzerland/

table/?disjunctive.name&disjunctive.abbreviation_canton_and_fl&sort=update (accessed 19 May 2022).
